# Clavicular Fractures in Newborns: What Happens to One of the Commonly Injured Bones at Birth?

**DOI:** 10.7759/cureus.18372

**Published:** 2021-09-29

**Authors:** Hina Mumtaz Hashmi, Nazia Shamim, Vinod Kumar, Noureen Anjum, Khalil Ahmad

**Affiliations:** 1 Pediatrics and Child Health, Aga Khan University Hospital, Karachi, PAK; 2 Obstetrics and Gynaecology, Aga Khan University Hospital, Karachi, PAK

**Keywords:** birth weight, erb's palsy, neonate, macrosomia, clavicular fracture

## Abstract

Introduction

The clavicle is one of the most commonly injured bones during the birth process. The objective of this study was to determine the frequency and outcome of fractured clavicle amongst neonates born in a five-year period at a Secondary Hospital setting and to determine the Maternal and Neonatal Characteristics involved in such cases and compare them with a control group and determine the significance of any factors.

Methods

All cases of fractured clavicle were retrospectively reviewed in a Secondary care hospital setting during a five-year period from July 2015 to June 2020. Maternal and neonatal factors were determined and then compared to a control group.

Results

Out of 21,435 live births at our center during the study period, 92 infants were diagnosed to have clavicle fractures, giving an incidence of 4.29 per 1,000 live births (0.43%). 89% cases (n=82) were detected before discharge and 11 % cases (n=10) on routine follow-up visit after discharge. Physical examination identified 77% cases (n=71) whereas 23% cases (n=21) were recognized incidentally on X-ray. All babies with fracture including 3 with Erb’s palsy recovered completely without any complications. On logistic regression analysis, spontaneous vaginal delivery, prolonged second stage, vertex presentation, vitamin D deficiency in mothers, birthweight, macrosomia, all were significant risk factors.

Conclusion

Neonatal clavicular fracture appears to be a transient yet unpredictable and unavoidable event with an overall good prognosis. Only the birth weight was identified as the common risk factor affecting clavicular fracture. Parental concerns and anxiety can be decreased with proper counselling and reassurance.

## Introduction

Clavicular fractures are the most commonly reported fractures in neonates [1]. These are birth-related fractures that may be avoidably occurring during the process of labor and delivery. The incidence of clavicular fracture in the newborn is between 0.2% and 4.5% [2]. The cause, of this condition, has not been precisely recognized and is considered an incidental and unpredictable finding on routine examinations of newborns [3]. The history of clavicular fractures dates back to 1764 when it was first reported by Erb W., along with Brachial plexus palsy and since then has been the focus of many publications with the aim of determining the cause and outcome with varied findings in literature review [4].

The occurrence of these fractures is related to many factors including maternal, fetal and skills of the obstetrician [5]. Prior studies suggest that the incidence of clavicular fractures amongst newborns may well be miscalculated due to the lack of consistent policies for screening in different health care setups [6]. Literature review reveals many studies over the incidence and risk factor leading to birth injury amongst neonates. In previous studies, many authors express hope and focus that improved obstetric techniques, including more frequent use of cesarean delivery might lower the occurrence of birth injury. This hope, however, has not been confirmed by previous studies [7]. Despite the association in most cases with traumatic delivery due to obstetric causes, most clavicle fractures occur in normal newborns after uncomplicated deliveries, and so it is an unpredictable complication [8]. In a prospective study done at Jordan, the incidence of clavicular fractures was 3.98/1000 [9], whereas in Malaysia an incidence of 0.64/1000 live births was observed [10]. Another 10-year study done in Japan shows 0.41% incidence of clavicular fractures amongst all live births [11]. In contrast, a study done in two teaching general hospitals in Pakistan showed an incidence of 1.4/1000 live births. Neonatal clavicular fracture is usually diagnosed by clinical examination as asymmetric Moro reflex, tenderness, swelling and crepitation on the affected site, and incidentally on radiography [6]. Clavicular fractures heal spontaneously without any long-term complications [12].

The aim of this study was to identify the incidence of clavicular fractures amongst newborns being born at a secondary care hospital and determining possible factors predisposing to this condition and developing strategies to prevent it so as to reduce anxiety and undue distress to the newborn and family. Since the literature shows a low incidence of this particular birth injury, a nested case-control would be the most appropriate design to identify such cases and compare with good number of controls.

## Materials and methods

Study design

After obtaining ERC approval, a nested case-control study design was used with a non-probability sampling strategy for both cases and control. A retrospective analysis was done of medical records of 92 neonates with fractured clavicles among 21,435 deliveries that occurred from July 2015 to June 2020 at Aga Khan Secondary Hospital for women, Karimabad. Physical assessment of the infant was done during the stay in the hospital at the time of delivery and on routine well-baby and nursery rounds. Radiography confirmed clavicular fracture. The maternal and infant files were reviewed and information collected on predefined Proforma. Information included maternal component (demographics, BMI, prenatal course, duration of labor, labor and delivery complications including shoulder dystocia and any associated maternal co-morbids like Gestational diabetes or vitamin D deficiency). Neonatal components included baby’s anthropometry, gender, Appearance, Pulse, Grimace, Activity, and Respiration (APGAR)’s, position, any associated diagnosis and duration of hospital stay. The details on the clavicular fracture group included time and cause of detection, side and site of clavicular fracture with its course along with any associated comorbid like Erb’s palsy. The follow-up data of cases were collected by parents on a telephonic conversation after taking verbal consent. The data on cases were compared to a control group of neonates in a ratio of 1:4 cases to control selected randomly from the five-year timeline reviewed. Information was compared between control and clavicular fracture groups. 

Statistical analysis

Data underwent descriptive statistical analysis using SPSS 21.0. Descriptive data including the mode of delivery, gender of the cases, the side of fracture and clinical presentation of fracture was presented as frequency (n) or percentage (%). Chi-square test was used for categorical variables and p-value was calculated by chi-square test for the maternal and neonatal risk factors linked to clavicular fractures. p-value <0.05 was considered significant. Data confidentiality was maintained at all times. No personal identifiers will be used in any reports or publication of the study.

## Results

Ninety-two (92) cases of clavicular fracture were identified out of 21,435 babies delivered in the five-year period of study from July 2015 to June 2020. The overall incidence was 0.43 % or 4.29/1,000 births. Of the total vaginal deliveries, 0.65% resulted in fracture. A total of 77.2% neonates with clavicular fracture were born via spontaneous vaginal deliveries, 17.4% were delivered via assisted vaginal delivery (forceps/vacuum) and 5.4% were born via cesarean section. Only four late preterm babies with fracture were delivered via vaginal delivery between 35 and 37 weeks of gestation. The remaining 96% neonatal fractures took place at term between 37 weeks and 40+6 weeks’ gestation. 58.7% mothers of babies with neonatal fracture were less than 30 years of age and 54% were obese with a BMI >30 kg/m^2^. 53.3% mothers were multipara. Although not a routine investigation for mothers in the antenatal period, Vitamin D levels amongst 43.8% cases were found deficient of the 34.5% cases of clavicular fracture who underwent vitamin D testing for other reasons. Amongst maternal co-morbids, gestational diabetes was seen in 31.5% cases and pregnancy induced hypertension in 6.5% cases respectively. 22.8% cases had shoulder dystocia. On reviewing neonatal factors, anthropometry of cases revealed 55% of infants with birth weight between 50th and 90th centile, 60% of birth height >95th centile and 54% of head circumference between 50th and 95th centile. Ten cases (10.9%) were macrosomic with a head circumference of >95th centile. Presentation at birth commonly seen was 94.5% cases as occipitoanterior (vertex) (Table 1).

**Table 1:** Maternal and neonatal factors of clavicular fractures. PIH: pregnancy-induced hypertension; BMI: body mass index; SVD: spontaneous vaginal delivery; Emlscs: emergency lower segment Cesarean section; Elscs: elective lower segment Cesarean section; APGAR: Appearance, Pulse, Grimace, Activity, and Respiration.

Features		n	%
Maternal factors			
Maternal age	<30 years	54	58.7
Parity	Multipara	49	53.3
BMI (kg/m^2^)	Mean	29.91	-
	Overweight (25-29.9)	31	33.6
	Obese (>30)	50	54
Maternal co-morbids	Vitamin D deficiency	14	15.2
	Gestational Diabetes	29	31.5
	PIH	6	6.5
Mode of Delivery	SVD	71	77.2
	Vacuum	14	15.2
	Forceps	2	2.2
	Emlscs	4	4.3
	Ellscs	1	1.1
Prolonged second stage		3	3.3
Gestational age (weeks)	Mean	38.1	-
	Late preterm (< 37)	4	4
	Term (37-40^+6^)	88	96
Shoulder dystocia		21	22.8
Neonatal factors			
Gender	Male	43	47
	Female	49	53
APGAR <7	At 1 min	9	9.8
	At 5 min	1	1.1
Birth weight	50^th^ – 90^th^ centile	51	55
	>3500 grams	34	37%
Birth height	>95^th^ centile	55	60
Birth head circumference	50^th^ – 90^th^ centile	50	54
Macrosomia	>95^th^ centile	10	10.9
Presentation	Occipitoanterior	87	94.5
	Occipitoposterior	1	1.1
	Breech	1	1.1
	Compound	2	2.2
	Unstable	1	1.1
Associated diagnosis	Meconium stained liquor	6	6.5
Duration of hospital stay	<48 hours	87	95
	Mean (Hours)	40	-

**Figure 1:**
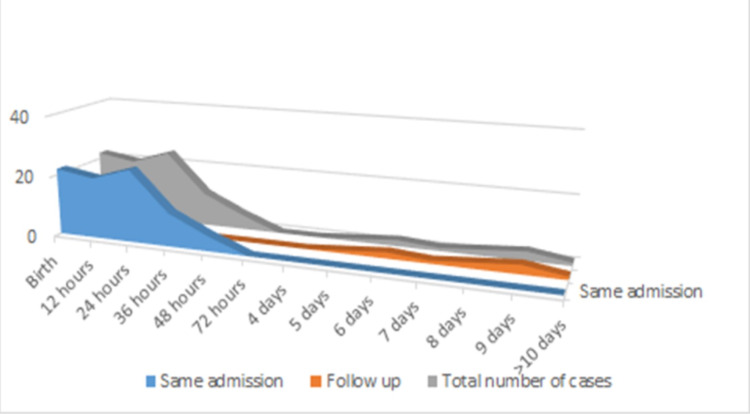
Graph showing time of diagnosis of cases in total, during same admission and on follow-up.

The right clavicular fracture was observed more than the left occurring in 55 cases (60%) and 56 cases (61%) showing fracture site at the mid-shaft of the clavicle. 89% of cases were diagnosed before discharge with 24% detected at birth, 48% within 24 hour of life, 12% and 5% at 36 and 48 hours of life. A graphical presentation showing the time of diagnosis of cases demonstrated majority (73%) were diagnosed within 24 hours of birth (Figure 1).

Ten cases (11%) had fracture detected after discharge on follow up. Among the cases, 71 infants (77%) had findings on presentation and examination in the form of crepitus over clavicle (57%), swelling over clavicle with decreased arm movement (20%), swelling over clavicle (15%), and incomplete Moro’s reflex (10%), whereas 23% cases were detected as incidental finding on X-ray for evaluation of respiratory symptoms and other reasons. Three infants (3%) presented with Erb’s palsy but without any sequel (Table 2).

**Table 2:** Clinical feature and outcome of clavicular fracture.

Feature		n	%
Side of fracture	Right	55	60
	Left	37	40
Site of fracture	Medial 1/3	8	8.6
	Midshaft	56	61
	Lateral 1/3	28	30.4
Time of diagnosis	Before discharge	82	89
	After discharge	10	11
Time of detection before discharge	Birth	22	24
	24 hours	44	48
	36 hours	11	12
	48 hours	5	5
Cause of diagnosis	Symptoms and examination	71	77
	Incidental on X-ray	21	23
Clinical presentation	Decreased upper limb movement	18	20
	Incomplete Moros	9	10
	Crepitus over fracture	52	57
	Swelling over fracture	14	15
Brachial plexus injury		3	3
Prognosis	Complete recovery	92	100
Follow-up	Pediatrician	78	84.8
	Orthopedic	9	9.8
Repeat X-ray		21	22.8

Ninety-two cases were compared at a ratio of cases: control; 1:4 to a group of 369 babies without clavicular fracture. Maternal factors such as age, parity, gestational age were not statistically significant. Mainly mothers in both group were < 30 years of age (58.7% vs 58.8%). The mothers in the fracture group were commonly seen to have undergone spontaneous vaginal delivery. (77.2% vs 52.8%). Female preponderance was seen in both group (53% and 55%). Macrosomic babies with a head circumference of more than 95th centile was observed in the fracture group compared to the control group (10.8 vs 4%). Analysis revealed birth weight (p-value <0.05), prolonged second stage (<0.008), vaginal delivery (<0.001) and macrosomia (<0.014) as significant risk factors for clavicular fracture with a statistically significant p-value of less than 0.05 (Table 3).

**Table 3:** Comparison of factors and significance between fracture and control group. SVD: spontaneous vaginal delivery.

Factors			Fracture group, n=92	Control group, n=369	p-value
Maternal age		<30 years	54 (59%)	217 (59%)	0.538
		>/= 30 years	38 (41%)	152 (41%)	
Parity		Primigravida	43 (47%)	140 (38%)	0.078
		Multipara	49 (53%)	229 (62%)	
Mode of delivery		SVD	71 (77%)	195 (53%)	<0.001
Presentation		Vertex	87 (95%)	360 (98%)	0.002
Prolonged second stage			3 (3%)	0	0.008
Gestational age		37-40+6 weeks	88 (96%)	325 (88%)	0.141
Gestational diabetes			29 (32%)	96 (26%)	0.175
Vitamin D deficiency			14 (15%)	62 (17%)	<0.000
Gender		Male	43 (47%)	204 (55%)	0.088
		Female	49 (53%)	165 (45%)	
Macrosomic			10 (11%)	15 (4%)	0.014
Birth weight	50^th^ – 90^th^ centile		51 (55%)	102 (28%)	0.000
	Mean (grams)		3282	2909	

A comparison was done between factors in cases diagnosed before discharge and those picked on follow-up showing most cases diagnosed before discharge had crepitations on physical finding (60.9%), and occurred on the right side (60.9%) as compared to cases picked on follow up. In comparison, the majority of cases on follow-up were born via spontaneous vaginal delivery (90%), were males (60%) and the fracture site was mid-shaft (80%) (Table 4).

**Table 4:** Comparison of factors between cases diagnosed in same admission and those on follow-up. SVD: spontaneous vaginal delivery.

Factors		Number of cases	
		Diagnosed in same admission, n=82 (%)	Diagnosed on follow-up, n=10(%)
Maternal co-morbid	Gestational diabetes mellitus	26 (31.7)	3 (30)
Prolonged second stage		3 (3.65)	0
Gestational age	Preterm (<37 weeks)	3 (3.65)	0
	Post-dates (>41 weeks)	1 (1.2)	0
Mode of delivery	SVD	62 (75.6)	9 (90)
Shoulder dystocia		19 (23.17)	2 (20)
Incidental		11 (13.4)	10 (100)
Gender	Male	37 (45)	6 (60)
Physical signs	Decreased upper limb movement	18 (21.9)	0
	Incomplete Moro’s	9 (10.9)	0
	Swelling	13 (15.8)	1 (10)
	Crepitation’s	50 (60.9)	2 (20)
	Erb’s palsy	3 (3.65)	0
Birth height	>95^th^ centile	48 (58.5)	7 (70)
Macrosomia		10 (12)	0
Fracture side	Right	50 (60.9)	5 (50)
Fracture site	Mid-shaft	48 (56)	8 (80)
	Lateral	28 (23)	0
	Medial	6 (7.3)	2 (20)
Assessed by orthopedic		9 (10.9)	0
Repeat X-ray at 3 months		19 (23)	2 (20)

84.8% of cases had a follow-up with a pediatrician and 9.8% of cases visited the orthopaedic once. Mean hospital stay was 40 hours, with 95% of cases discharged within 48 hours of life. The recovery was 100% with no complications.

## Discussion

Birth injuries can occur as a result of the stressful process and physical force during delivery and labor. Clavicular fractures are the most frequent injury experienced by newborns during birth. It is usually unpredictable and commonly encountered in spontaneous vaginal delivery. Good birth weight is one of the predisposing factors. Since clavicular fractures in neonates resolve spontaneously with no long-term complication, parental reassurance and soft handling are required for management. Assisted deliveries like forceps were done in the past to help pull fetus from high in the birth canal. This led to an increased incidence of birth injuries in the past. Today forceps and vacuum are normally used near-final stages of delivery [13].

The clavicle is the commonest bone fractured during stress of birthing process. Our study identifies incidence to be 4.29 cases/1,000 births similar to the review showing 6.5, 4.11, 1.96 and 3.97 cases of fracture per 1,000 live births [3,11,12]. The variability in incidence could be due to associated risk factors such as birth weight, birth head circumference, mode of delivery, parity of the mother, gestation of the infant, shoulder dystocia, birth presentation and expertise of delivery [11,13].

Literature suggests vaginal deliveries to be more correlated to the occurrence of clavicular fracture (77.8% and 61.5%) [9] [10]. This is similar to as seen in our study (77.2%). In contrast, a 10-year literature review reveals only a 4.4% affiliation of cesarean deliveries to clavicular fracture similar to seen in our study, 5.4% [11]. Forceps and vacuum delivery were seen to be present in 15% and 23% cases as compared to 2.2% and 15.2% in our study, respectively.

As suggested in the former literature review the right clavicle is predominantly involved; 69.6% [11] and 59% [3] as seen in our study 60% as compared to 40% fractures involving the left side. No case of bilateral clavicle involvement was seen in our study. Most cases involved mid-shaft (61%) as compared to a review of fourteen cases of clavicular fracture in which all the fractures were mid-shaft in position [10].

Fractures of the clavicle maybe displaced or non-displaced. The latter ones are usually asymptomatic and can be picked up on follow-up as well after formation of callus and healing of the fractured part [14]. A study reported up to 40% of cases being picked on follow-up after discharge from the hospital [15]. This was in contrast to 11% of cases in our study which were picked on follow-up. A thorough and repeated clinical examination at the time of birth and during well-baby rounds can be the reason for the timely detection of clavicular fracture before discharge.

23% of cases were diagnosed incidentally as compared to 54.9% in a 10-year study review [11]. In a five-year retrospective review, the clinical presentation of decreased upper limb movement, incomplete Moro’s, crepitus and swelling over the fracture were seen in 30.9%, 1.1%, 31.4% and 13.8% respectively [3]. Similar numbers were reported in our study except for incomplete Moro’s and crepitus which was present relatively more in our study; 10% cases demonstrating incomplete Moro’s and 57% cases presenting with crepitus. Erb’s palsy was seen in 3% of cases in our study, half of this number was seen in our reference study, 1.6% [11].

When comparing factors in the fracture and control group, the birth weight was noted to be statistically significant in our study (p-value - 0.000) similar to other studies in which birth weight was one of the consistent significant factors noted. [11,15,16] Macrosomia was also noted to be significant (p-value: 0.014). Some literature supports our findings [15,16] while in others macrosomia was not found to be statistically significant [3]. A prolonged second stage, spontaneous vaginal delivery, vertex presentation and vitamin D deficiency were other significant factors noted whereas maternal age, parity, gestational diabetes, gestational age and gender of the baby were not statistically significant similar to another study [11].

Some studies suggest incidence of clavicular fracture as an indicator of quality control. Although studies show a greater incidence in vaginal deliveries, cesarean section to avoid this injury is not recommended [3]. This study, like previous studies, points out that healthcare workers need to do a thorough examination even after discharge.

## Conclusions

Our study confirms that most cases of clavicular fractures In newborn babies are associated with spontaneous vaginal delivery while the delivery through cesarean section does not make a baby immune to the occurrence of this birth injury. Diagnosis is usually clinical but can be incidentally made and confirmed by radiological evaluation. The outcome is excellent with no neurologic sequel. Various maternal and neonatal factors were identified although apart from birth weight no other factor was seen to be consistently significant in the literature review thus making it difficult to predict most cases of clavicular fractures. No specific prevention could be suggested except a thorough examination both before and after discharge to detect any asymptomatic cases.
